# Biofilm formation is a risk factor for late and delayed complications of filler injection

**DOI:** 10.3389/fmicb.2023.1297948

**Published:** 2024-01-08

**Authors:** You-liang Zhang, Zhong-sheng Sun, Wei-jin Hong, Yin Chen, Yang-fan Zhou, Sheng-kang Luo

**Affiliations:** ^1^The Second School of Clinical Medicine, Southern Medical University, Guangzhou, Guangdong, China; ^2^Department of Plastic and Reconstructive Surgery, Guangdong Second Provincial General Hospital, Guangzhou, Guangdong, China; ^3^Department of Health Management, Guangdong Second Provincial Genera Hospital, Guangzhou, Guangdong, China; ^4^Department of Pathology, Guangdong Second Provincial General Hospital, Guangzhou, Guangdong, China

**Keywords:** biofilm, soft tissue filler, hyaluronic acid, complications, histopathology

## Abstract

**Introduction:**

Biofilm formation is a major cause of delayed-graft complications. Similarly to implants, dermal fillers carry the risk of biofilm formation, which can lead to the development of nodules, chronic inflammatory reactions, abscesses and other complications. In this study, we investigated the late or delayed complications associated with biofilm formation on dermal fillers.

**Methods:**

In this retrospective analysis, we analyzed all cases of complications caused by filler injections at a single center between January 2017 and December 2022, the majority of which comprised nodule formation and chronic persistent inflammatory reactions. The risk of biofilm formation with fillers was summarized and analyzed based on the results of bacterial culture and pathological examination.

**Results:**

Sixty-one patients were enrolled, including 42 cases of nodule formation, 15 of chronic inflammatory reactions, and 4 of active infection. Bacterial culture of the tissue samples obtained from seven patients after surgical treatment were positive, and comprised four cases of *Staphylococcus aureus*, one case of *Staphylococcus epidermidis*, one case of *Staphylococcus saprophyticus* and one case of *Mycobacterium abscessus*. The corresponding histopathological results indicated extensive mononuclear lymphocyte infiltration, with a giant cell reaction in the fibrous connective tissue.

**Conclusion:**

The results of this study suggest that biofilm formation is a significant risk factor for late and delayed complications following filler injection, and is caused by the contamination of resident bacteria and recessive infection at the injection site.

## 1 Introduction

The field of medical cosmetology has rapidly developed over the past 20 years, and filler injections have become increasingly popular. This may be attributable to the precision of injection technology, the development of new injectable materials and the many advantages of this technique, including high efficiency and minimal invasiveness. However, with the increasing popularity of filler injections, complications have become hidden dangers that cannot be ignored (Ledon et al., 2013).

Several studies have suggested that late or delayed adverse events are caused by biofilm formation (Christensen et al., 2013; Alhede and Bjarnsholt, 2014). Biofilms are defined as a heterogeneous structure predominantly composed of bacteria embedded in an extracellular matrix, which can form on the surface of implants (Rohrich et al., 2010). The protein expression and characteristic variations of biofilms can lead to immune escape by reducing the growth rate and hindering the phagocytic action of macrophages (Costerton et al., 1999). Therefore, biofilms often show super-strong drug resistance (Proctor et al., 1998; Ceri et al., 1999). Biofilm formation on implants is often characterized by formation of a fibrous envelope, which can cause symptoms such as capsular contracture after breast augmentation. The fillers used for cosmetic injections, such as hyaluronic acid, a component of the biofilm extracellular matrix, also carry the risk of biofilm formation.

Biofilm-forming bacteria are generally opportunistic pathogens, and the resident flora of the skin and mucosa, such as Streptococcus, Enterococcus, and Staphylococcus, may contribute to biofilm formation (Toy and Frank, 2003; Ghislanzoni et al., 2006). During filler injection, when the needle inadequately penetrates sterilized skin, it allows resident bacteria to contaminate the injection site, allowing them to reside in the filler and form a biofilm as they proliferate. In addition, recent research has suggested that the adherence of bacteria fillers and bacteremia can both play a causative role in associated complications (Decates et al., 2023). Research has also identified a higher risk of biofilm formation in the presence of bacterial contamination during the injection procedure and latent infection at the injection sites, resulting in delayed complications, such as nodules and chronic inflammatory reactions with repeated and intractable symptoms (Alhede et al., 2014; Ibrahim et al., 2018). The above pathological processes may affect filler degradation, resulting in the non-resolution of symptoms as the filler slowly degrades. Delayed biofilm-related complications thus pose a significant challenge in the field of filler injections.

In this study, we reviewed all cases of late or delayed complications following filler injection, including nodule formation and chronic inflammatory reactions, which occurred at our center, and analyzed the possible mechanisms of biofilm formation related to filler injection through the investigation of clinical manifestations, bacterial cultures, histopathological observations, and treatment.

## 2 Materials and methods

We retrospectively reviewed the medical records of patients with late and delayed complications associated with cosmetic filler injections at our department (Department of Plastic and Reconstructive Surgery, Guangdong Second Provincial General Hospital, Guangzhou, Guangdong Province, China) between January 2017 and December 2022. This retrospective study conformed to the Declaration of Helsinki and was exempt from review by our institutional review board.

The inclusion criteria were as follows (Cassuto and Sundaram, 2013; Ibrahim et al., 2018): (1) Patients aged >18 years who experienced late or delayed complications following filler injection; (2) Patients who exhibited delayed symptoms related to filler injection, including erythematous, pain, papules or inert granuloma; (3) Patients who presented with a delayed active suppurated infection and abscess, which may have recurred after successful treatment; (4) Patients who presented with an infiltration of a large number of inflammatory cells around the filler with a giant cell reaction on histological examination, or a positive culture indicating biofilm formation. It is important to note that negative results from the above tests could not rule out the diagnosis.

Adherence to the inclusion criteria were determined through investigation of each patient’s histopathological findings and symptoms. The series comprised 61 patients, including 42 cases of nodule formation (68.9%), 15 cases of nodules accompanied by chronic inflammatory reactions (24.6%), and 4 cases of secondary local infection (7.5%). Symptoms of these complications did not appear until more than 3 months after the injection procedure and occurred at or close to the injection site. The types of fillers and results of the bacterial culture were summarized and analyzed. Patients who underwent surgical treatment were recorded as having clinical manifestations of the lesions explored during surgery. The tissue samples obtained from the surgery group were subjected to histopathological observation.

## 3 Results

### 3.1 Demographics

**Figure 1:**
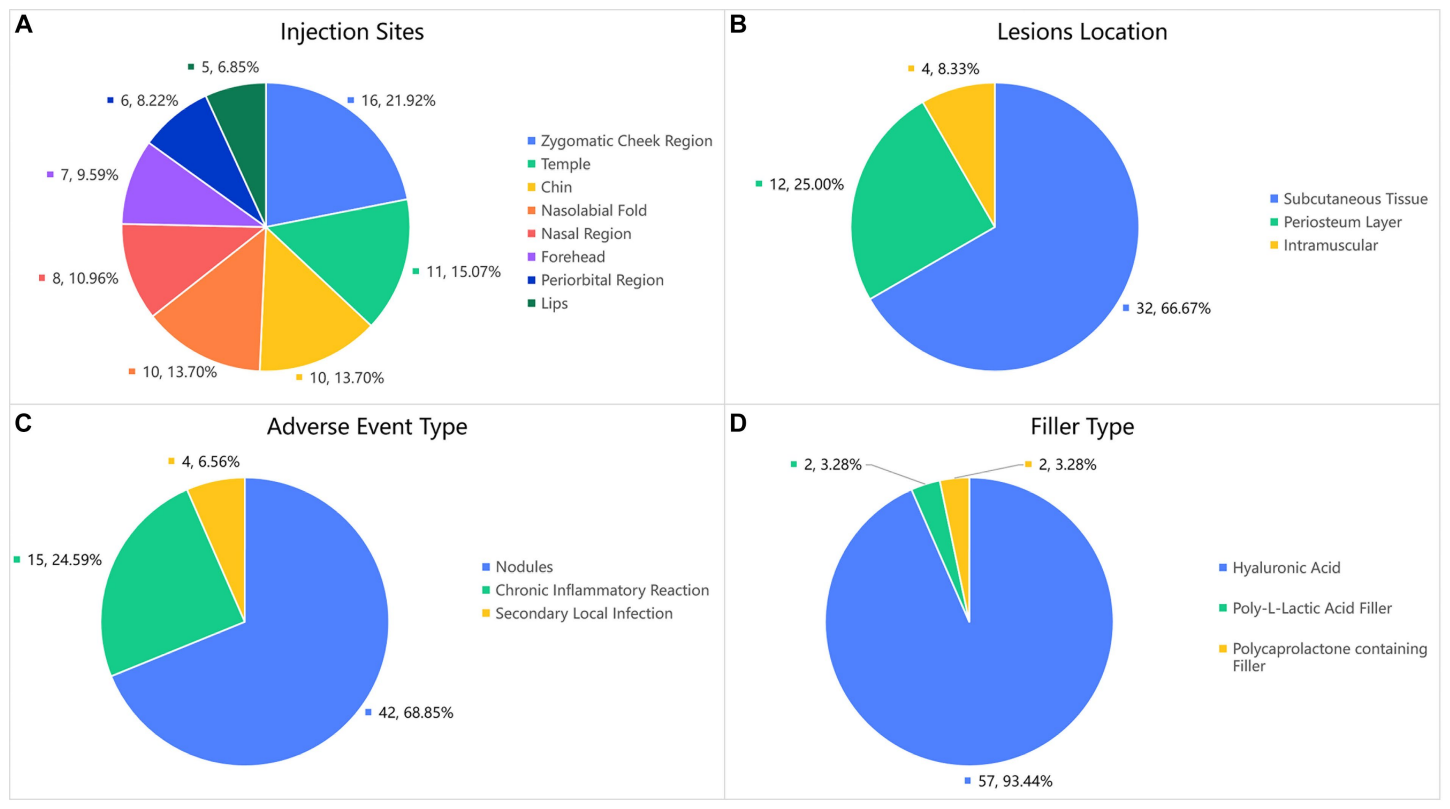
Demographics and clinical features.

The patient characteristics, including demographics and clinical features, are presented in Table 1; Figure 1. Among these cases, the average duration of late or delayed complications after filler injection was 16.2 months, with the shortest period of symptom onset was 3 months and the longest was 6 years after injection. In these cases, most of the injected filler was hyaluronic acid (57/61, 93.4%), and the others were 2 cases of Poly-L-Lactic Acid filler and 2 cases of Polycaprolactone microspheres containing filler. The site of symptoms was closely related to the injection procedure, with symptoms occurring at 73 sites in 61 patients. Among these, a high incidence of delayed complications was found in the zygomatic cheek region (16/73, 21.9%), temple (11/73, 15.1%), chin (10/73, 13.7%), nasolabial fold (10/73, 13.7%), nasal region (8/73, 11.0%), and forehead (7/73, 9.6%). Of the 48 patients who underwent ultrasound Doppler examination or surgical treatment, 32 (66.7%) of the lesions were found in the subcutaneous tissue, 4 in the intramuscular layer (8.3%) and 12 in the periosteum layer (25.0%). In addition, a total of 13 patients were injected repeatedly and/or with multiple fillers at the same site, among which eight (61.5%) developed an active infection or inflammatory reaction, accounting for a significantly higher proportion than the overall data.

**Table 1:** Demographics and clinical features.

Characteristics		Number/proportion
Gender	Female	59, 96.7%
	Male	2, 3.3%
Age, yrs., mean (SD)		38.3 (10.5)
Smoking habit	Yes	6, 9.8%
	No	55, 90.2%
Filler type	Hyaluronic acid	57, 93.4%
	Poly-L-Lactic Acid Filler	2, 3.3%
	Polycaprolactone containing filler	2, 3.3%
Time for complications after filler injection, months, mean(SD)		16.2 (12.6)
Injection Sites (73 sites in 61 patients)	Zygomatic Cheek Region	16, 21.9%
	Temple	11, 15.1%
	Chin	10, 13.7%
	Nasolabial fold	10, 13.7%
	Nasal region	8, 11.0%
	Forehead	7, 9.6%
	Periorbital region	6, 8.2%
	Lips	5, 6.8%
Lesions location	Subcutaneous tissue	32, 66.7%
	Periosteum layer	12, 25.0%
	Intramuscular	4, 8.3%
Adverse event type	Nodules	42, 68.9%
	Chronic inflammatory reaction	15, 24.6%
	Secondary local infection	4, 7.5%

### 3.2 Symptoms, examinations, and treatments

Although filler nodule formation can also occur due to the accumulation of fillers, biofilm should be considered as a causative factor for local nodules that develop 3 months after injection. Nodules are usually accompanied by varying degrees of local tingling, mild swelling, rashes, or pruritus. Active inflammation or infection is not the original symptom; in fact, it is usually triggered by factors such as other underlying diseases, acute infection, the menstrual cycle and mental stress. After symptoms occur, oral medication can provide temporary relief; however, symptoms recur when the medication is discontinued. In this study, 41 patients (67.2%) had one of these symptoms, 27 (44.3%) had two, and 19 (31.2%) had three or more recurrent local symptoms.

**Figure 2:**
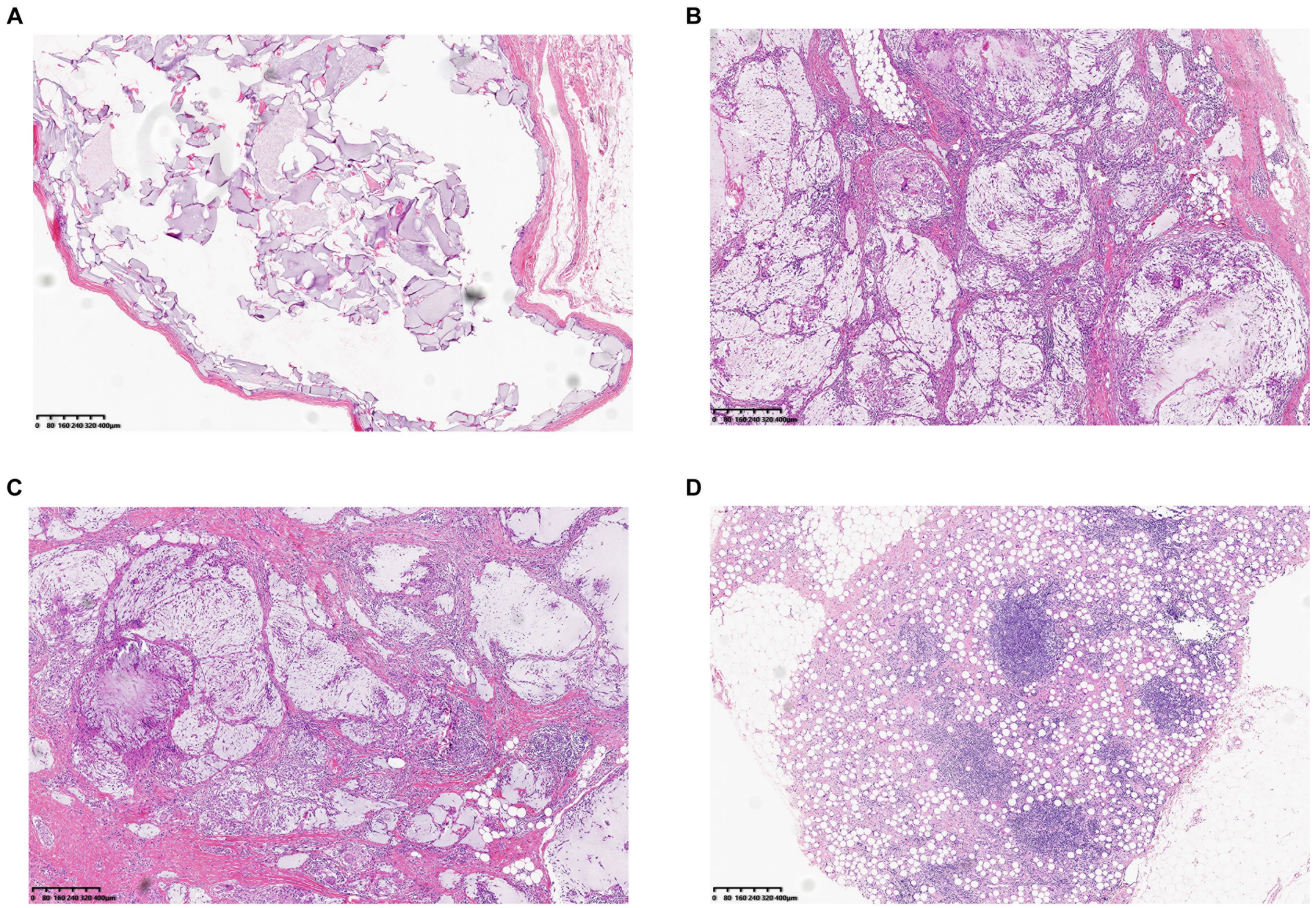
Two distinct histopathological findings of biofilm-associated nodule: **(A)** Histological findings showing infiltration of monocytes around the filler, the fibrous envelope is thin, and the shape of the filler is relatively intact. **(B,C,D)** Nodules accompanied by repeated inflammation and abundant monocyte infiltration in and around the filler, accompanied by a giant cell reaction (Filler type, **B**, **C** Hyaluronic acid. **D** Polycaprolactone containing Filler).

In terms of treatment, hyaluronidase is the preferred option in the cases of hyaluronic acid injection. Easing the filler is essential to avoid recurrence of local symptoms. Except for cases of chronic inflammatory reactions and secondary local infection, 35 patients received local hyaluronidase application. Nodules that did not respond to hyaluronidase treatment were treated with intralesional corticosteroid injections. For patients with chronic inflammatory reaction and local infection, treatment also include glucocorticoids and antibiotics (typically quinolones and macrolides). When symptoms recur and severe local active inflammation or infection occur, conservative treatment is unsatisfactory, and surgery is the final option. In our cohort, 18 patients underwent surgery. Tissue samples taken during surgery were cultured for bacteria, seven of which were positive, including four cases of *Staphylococcus aureus*, and one case each of *Staphylococcus epidermidis*, *Staphylococcus saprophyticus*, and *Mycobacterium abscessus*. Histopathological examination (Figure 2) showed two distinct manifestations. For nodules without local symptoms, the histological manifestations included accumulation of filler within the fibrous envelope and local inflammatory cell infiltration only around the filler, which was not obvious. However, nodules with local symptoms (flushing, swelling, pain, pruritus, and rash) showed histological manifestations of massive mononuclear infiltration in and around the filler, accompanied by granulomatous inflammation and giant cell reactions, which are significantly more severe. The histological characteristics of these inflammatory granulomas are similar to those of capsular contracture following breast augmentation and are consistent with biofilm formation.

### 3.3 Clinical cases

#### 3.3.1 Case 1

**Figure 3:**
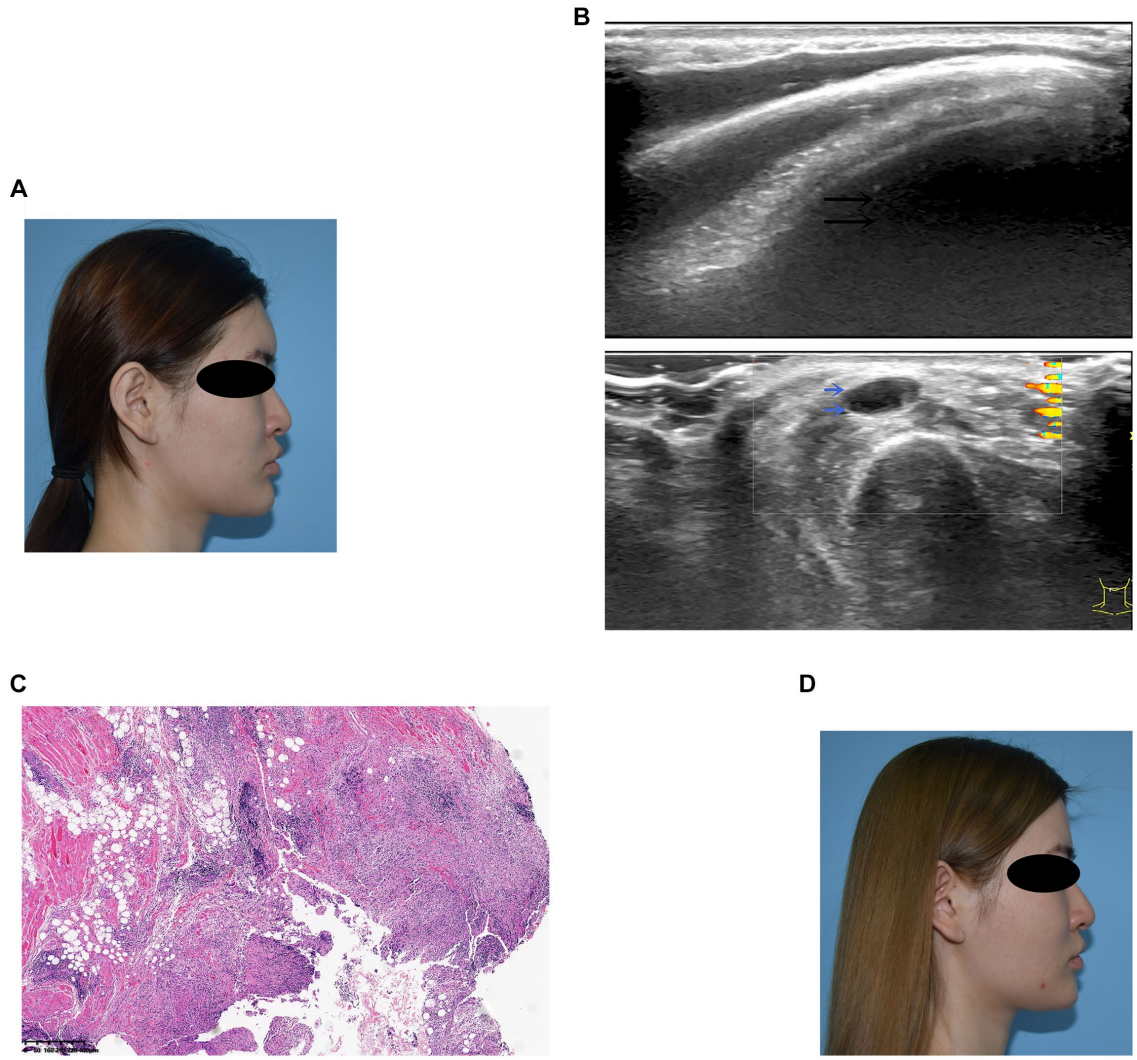
**(A)** Preoperative photograph showing swelling of the forehead and multiple nodules on the lips and chin. **(B)** Ultrasound examination: The black arrow denotes the fluid-echoless area of the forehead, indicating a cystic lesion caused by a local active infection. The blue arrows indicate multiple hypoechoic nodules on the lips and chin. **(C)** Histological observation: The filler was accompanied by the infiltration of macrophages and giant cells. **(D)** Six months after surgery, the patient’s symptoms resolved.

A 23-year-old woman received filler injections in the forehead, lips, and chin 1 year prior. Six months after the injection, multiple nodules were found in the chin region, accompanied by persistent redness, swelling, and pricking on the forehead. The symptoms did not respond well to oral antibiotic treatment. Preoperative ultrasonography revealed a fluid-echoless area in the subcutaneous layer of the forehead and multiple low-echo nodules with clear borders on the lip and chin. Surgical management involved nodule excision and drainage. Two granulomas were removed from the chin, and viscous pus was drained from the forehead. Examination of tissue samples revealed no positive results for bacterial culture, and histopathological observations showed that the filler was surrounded by numerous macrophages, with a foreign body giant cell reaction. After surgery, the patient’s symptoms resolved. Images of this patient are shown in Figure 3.

#### 3.3.2 Case 2

**Figure 4:**
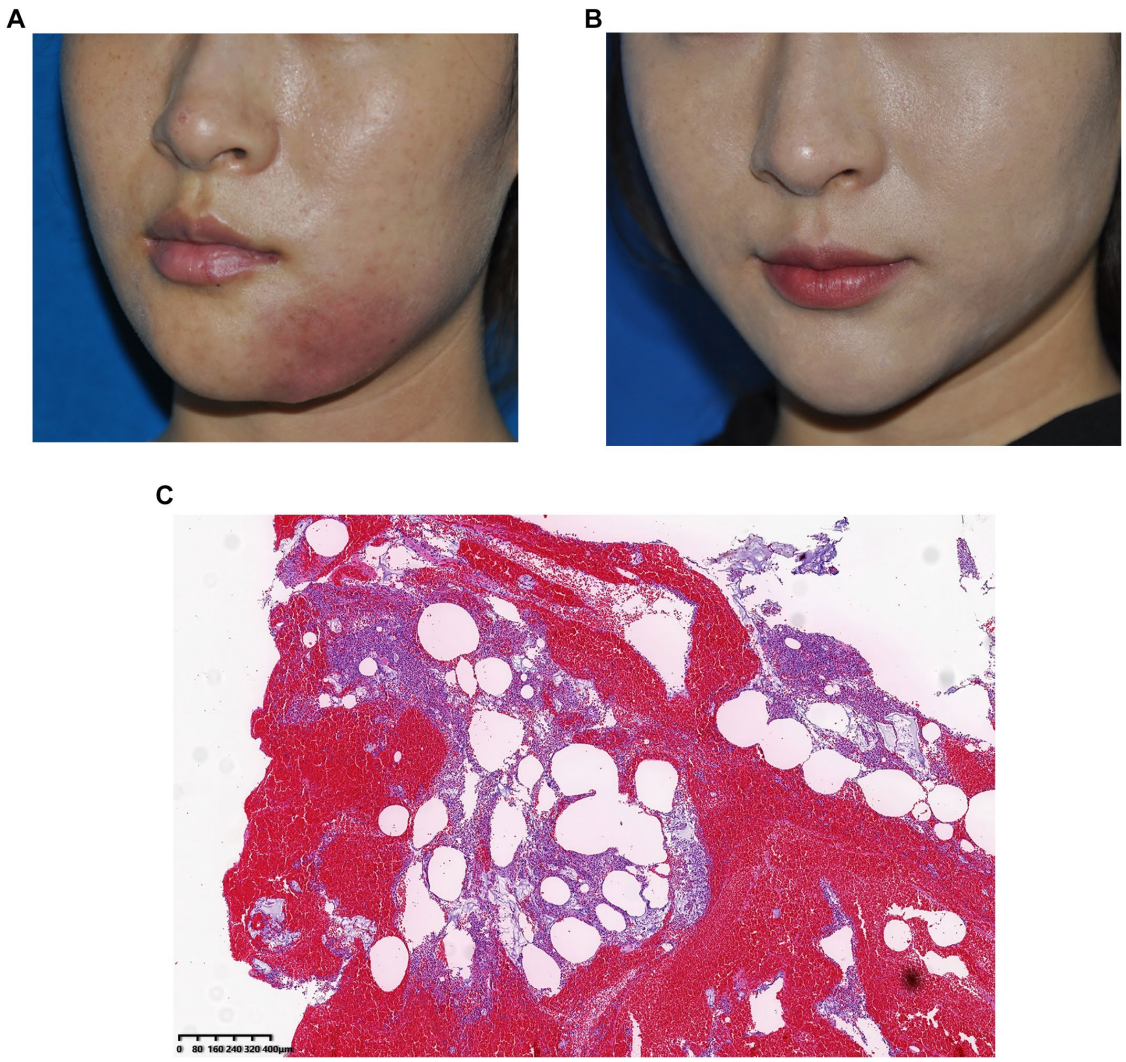
**(A)** Preoperative photograph, local infection of the mandibular region 1 year after hyaluronic acid injection and **(B)** Photograph taken 3 months after surgery. **(C)** Histological observation: The pus was doped with amorphous filler and a large number of monocytes infiltrated, indicating a severe inflammatory reaction caused by *Staphylococcus aureus* infection.

A 25-year-old woman received filler injections in the chin and mandibular regions. Three months after injection, the patient experienced swelling and tingling at the injection site. The symptoms gradually eased with oral antibiotic treatment but occasionally recurred. However, the symptoms worsened with local infection. During surgery, some fillers and pus were drained from the jaw. The bacterial culture was positive for *Staphylococcus aureus*. The patient’s symptoms resolved postoperatively. Images of this patient are shown in Figure 4.

#### 3.3.3 Case 3

**Figure 5:**
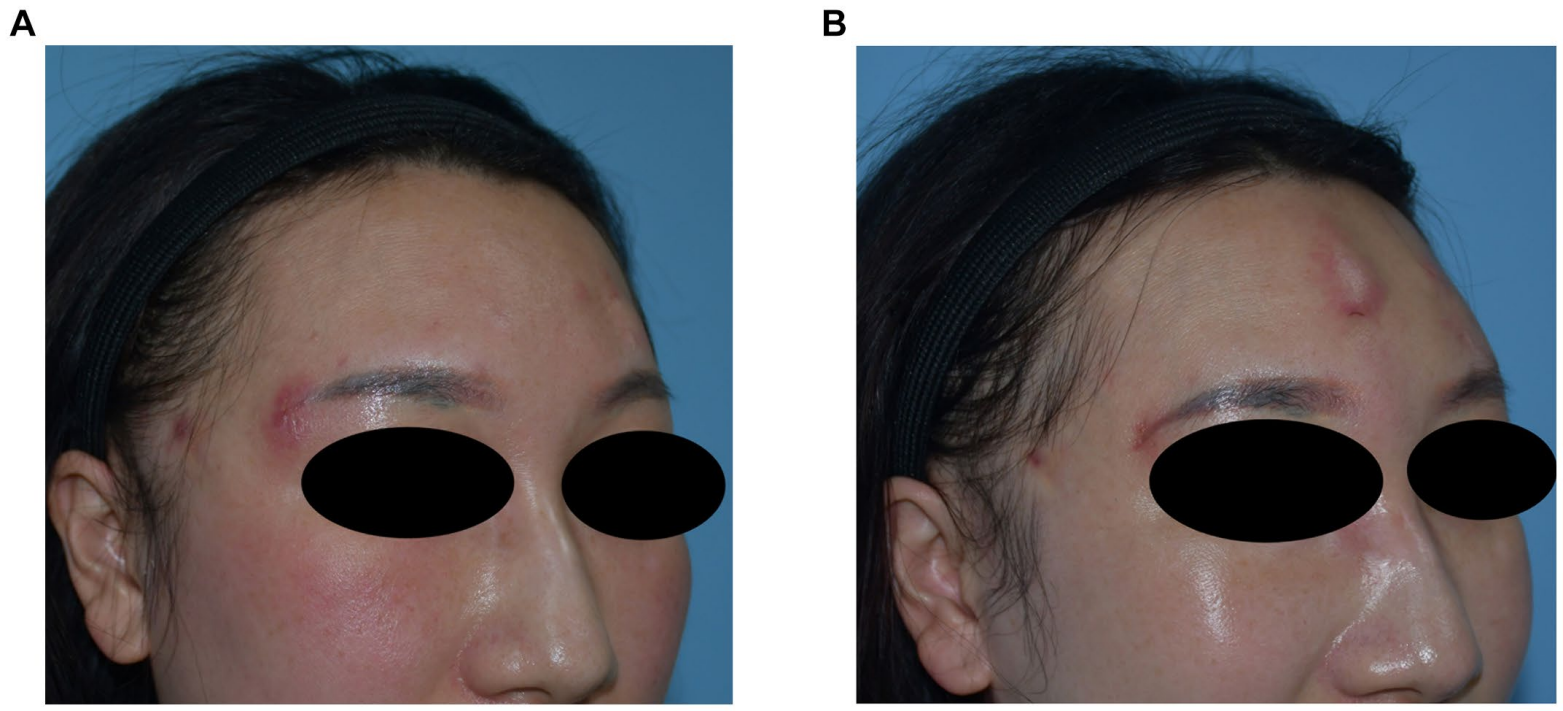
**(A)** Preoperative photograph showing multiple nodules in the forehead with recurrent local inflammation. **(B)** Recurrence of local symptoms after surgery is consistent with the characteristics of *Mycobacterium abscess* infection.

A 37-year-old woman developed nodules in the forehead and temple 10 months after hyaluronic acid injection. During this period, redness and swelling occurred repeatedly and did not respond to oral antibiotic treatment. Preoperative ultrasonography revealed multiple hypoechoic areas in the subcutaneous layer of the forehead and temple, along with swelling of the surrounding soft tissue. The filler and pus were removed and drained surgically; however, the symptoms recurred. Bacterial culture involving a special examination confirmed *Mycobacterium* abscess infection. After the patient underwent anti-tuberculosis treatment, the symptoms gradually resolved. Images of this patient are shown in Figure 5.

## 4 Discussion

The widespread use of cosmetic filler injections has led to an increase in the reported number of long-term complications. Late and delayed complications such as nodules, granuloma formation, and/or active infection are becoming increasingly problematic. Initially, these delayed complications were thought to be related to an immune response to the filler. However, the fibrous reaction of hydrophilic fillers, such as hyaluronic acid, is minimal, which is not consistent with granuloma formation after injection. As research has advanced, increasing evidence has been uncovered to suggest the presence of bacteria on the surface of implants, which means that biofilm formation has become the leading cause of device-associated infections in medical devices and implants. Over time, bacteria not only enter the filler material but also infiltrate the extracellular matrix, forming heterogeneous structures with high resistance and stability (Anwar et al., 1992; Rohrich et al., 2009; Singh et al., 2009). The bacterial load of the resulting heterogeneous structure can be extremely high, allowing positive results for bacterial testing of tissue samples (Costerton et al., 1999). *In vitro* studies have shown that some fillers support bacterial growth when contaminated. As a component of the extracellular matrix, hyaluronic acid has been proven to promote biofilm formation in contaminated conditions (Saththianathan et al., 2017). Injectable fillers containing hyaluronic acid pose a potential risk for biofilm formation.

Christensen et al. (2005) were the first to detect bacteria in tissue samples of filler-associated granulomas. Histological analysis of the tissue samples showed that the degraded filler particles were embedded in the fibrous tissue along with inflammatory cells, macrophages, and giant cells. Thus, the histological findings of the resected tissue samples were consistent with the clinical symptoms. In cases of recurrent inflammation, the histological results are distinct from those of inert nodules. Recurrent symptoms are accompanied by high concentrations of bacteria in and around the filler, leading to severe monocyte infiltration and giant cell reactions. The clinical outcomes of these patients are more complex, and treatment is more difficult.

Although filler injection is a minimally invasive procedure in clinical practice, there are certain risks associated with its performance. In some injection methods, multiple, multi-site, and multi-layer injections are required to achieve the ideal effect which certainly increases the risk of filler contamination due to repeated skin penetration (Saththianathan et al., 2017). In addition, potential infections at or near the injection site, or local infections resulting from medical procedures such as oral surgery are also risk factors for filler contamination. Moreover, the bacterial culture results obtained from histological samples exhibit variations compared to the resident skin microbiota, suggesting that bacteremia could be also a high-risk factor for uniformed patients (Decates et al., 2023).

The infectious bacteria found in delayed complications are usually opportunistic pathogens such as *Pseudomonas* and *Propionibacterium*, and resident bacteria of the skin and mucous membranes, such as *Staphylococcus* and *Streptococcus* (Saththianathan et al., 2017). This is consistent with the results obtained in our study. Specific symptoms such as recurrent active infections were highly related to positive bacterial cultures during long-term follow-up. The clinical symptoms of these bacterial infections are generally more insidious and recurrent, which is also consistent with the symptoms of these patients (Bjarnsholt et al., 2009).

The midface, including the nose, zygomatic-cheek region, chin, nasolabial fold, and periocular region, was the most prone to delayed complications in our study, which is consistent with the results of previous studies (Decates et al., 2023). In addition, there is a higher risk of late and delayed complications in some regions with larger injection volumes or those requiring mass injection, such as the temple, forehead, and chin. The injection layer of the above sites is deep, which is conducive for the hiding of bacteria. Gradual formation of biofilms in the perioral area could be associated with incomplete disinfection and bacterial contamination when the needle penetrates through the mucous membrane. Even if the injections are administered in the nasolabial fold, especially deep in the periosteum, an incorrectly performed procedure may allow the needle to come into contact with the mucous membrane on the oral side, thereby increasing the risk of bacterial contamination. Cases of biofilm formation have a high incidence of nodules at anatomically specific sites such as the periorbital region. For example, tear trough injections tend to involve the injection of fillers into the periosteum layer to form a bolus. Some active symptoms of biofilm formation on these fillers can be prominent due to the thin skin and soft tissue. Furthermore, our findings from the ultrasound examination indicate a significantly elevated risk of late and delayed complications in the subcutaneous tissue when compared to other layers. The subcutaneous tissue is an important site where local reactions following filler injection can manifest with increased severity. This phenomenon is closely linked to the inflammatory response, which is mediated by the infiltration of monocyte macrophages. Secondly, displacement of the subcutaneous facial tissue layer, resulting from the activity of facial muscles, introduces inherent instability. This instability can negatively affect the stability of fillers, leading to increased late and delayed complications.

*In vitro* studies have shown that repeated injections lead to a higher risk of bacterial contamination of the filler (Saththianathan et al., 2017). In fact, compared with patients who underwent a single filler injection, patients who received multiple injections, multiple types of fillers, or repeated injections after hyaluronidase treatment, were more likely to experience recurrence and severe symptoms. This group of patients were managed with a complex treatment course, including the application of hyaluronidase and corticosteroids, and even surgical excision, which could affect facial morphology, triggering a new need for filler injections. However, re-injection is a high-risk factor as biofilm formation is likely to occur and cannot not be treated using the previously described methods. The biological characteristics of the biofilm and the results of our histological examination support the difficulty of clinical treatment. The distribution of bacteria in the tissue and filler appears to be dense, and the infiltration of monocytes also indicates repeated and severe inflammatory reactions, which would undoubtedly render radical treatment extremely difficult.

In terms of prevention, we emphasize the importance of pre-injection disinfection. In previous research on nipple-areolar complex management, it was found that pre-disinfection had a positive effect on reducing the resident flora as well as a certain preventive effect on capsular contracture, which is related to biofilm formation (Zhang et al., 2021). The same is true for facial filler injections. The operator should not only perform good disinfection but should also pay attention to sterility during the injection process. In case of multiple injections in one region, disinfection before injection is not sufficient for the entire process. Therefore, we recommend the use of alcohol gauze during the process as it can re-disinfect the injection site during auxiliary procedures such as wiping and massaging, thereby reducing the risk of contamination. In addition, the needle passes through not only the skin but also appendages such as follicles and sebaceous glands that cannot be sterilized. Therefore, the needle could become contaminated during penetration. Furthermore, improper needle placement during injection increases the risk of bacterial contamination. These aforementioned risks can be prevented by changing the needle at the right time, which can also improve patient comfort during injection.

Clinical management of late and delayed complications is often phased (Cassuto and Sundaram, 2013; Ibrahim et al., 2018). The first step involves determining the type of filler causing the complications. Conservative treatment is generally acceptable for patients with inert nodules. Most nodules with delayed complications are granulomas; hyaluronidase treatment is often less effective than expected for these nodules. Even with ultrasound-guided hyaluronidase injection, the remaining granuloma carries the risk of biofilm formation, possibly triggering symptom recurrence. Nevertheless, hyaluronidase continues to be the preferred option for initial treatment. In instances in which hyaluronic acid is used as a filler, local administration of hyaluronidase is of particular significance. Hyaluronidase has also been reported to be effective in treating calcium hydroxylapatite granulomas (Bailey et al., 2011). Given the minimal risks associated with hyaluronidase, it may also be deemed suitable for the treatment of complication associated with alternative types of fillers. Simultaneously, the utilization of antibiotics is also imperative. In cases that local administration of hyaluronidase and antibiotic therapy prove to be ineffective, the administration of intralesional corticosteroids is recommended for patients (Lemperle et al., 2006; Beleznay et al., 2015).

For patients with local inflammatory reactions or symptoms of infection, the effect of conservative treatment is not ideal. The high bacterial load in the biofilm, and the subsequent persistent chronic inflammatory reactions are the causes of repeated symptoms. This condition also occurs in cases of capsular contraction after breast augmentation, for which excision of the capsule may be the most effective treatment. Therefore, surgery should be the most direct treatment for lesion removal. Preoperative ultrasonography can confirm the layers and boundaries of the nodules. For nodules in the superficial layer, skin incision was straightforward. It is not necessary to blindly avoid skin incision because exploration through a mucosal incision is difficult, with a higher risk of contamination. For nodules, the granuloma should be removed along with the capsule, whereas for inflammation or local infection, the lesion should be removed as thoroughly as possible. In most cases, incision and drainage were insufficient, and the infected and necrotic tissue was cleared under direct vision during the operation and repeated irrigation of the cavity with an antibiotic solution. All tissue samples and pus obtained required histopathological examination and bacterial culture, and postoperative treatment was adjusted based on the examination results.

Although the mechanisms of late and delayed filler-related complications are complex, biofilms are an important cause. However, clinical treatment is difficult. Therefore, preventing implant and filler contamination is important to avoid late and delayed biofilm-related complications. In addition to strict disinfection of the injection site, several other considerations should be noted: (1) The skin should be selected as the injection site rather than the mucosa. (2) Needle changes are recommended for regions requiring large injection volumes or multiple injections. (3) Patients with inflammation, acne, or allergic reactions at the injection sites should not receive fillers. (4) Repeated injections of various fillers into a single region are not recommended. (5) Additional care must be taken when performing reinjections in areas where late and delayed complications have previously occurred.

This study has several limitations that should be mentioned. As a single-center retrospective study, the small sample size may lead to data bias. In addition, histological examination can only show the pathology of the filler and tissue but cannot confirm the presence of bacterial infection, especially if the positive rate of bacterial culture is not high.

## 5 Conclusion

Overall, the results of this study confirm that biofilm formation is a neglected but significant risk factor for late and delayed complications of filler injection, and that it is related to the contamination of resident bacteria and recessive infection of the injection site. Biofilm formation is often insidious; some clinical cases present with no obvious inflammatory reactions on histological observation of inert nodules. Under certain conditions, inert nodules can be transformed into chronic inflammatory reactions and even severe local infections due to the high load of bacteria. Clinical management of these complications is complex and difficult. Therefore, prevention of the complications is more important than treatment.

## Data availability statement

The original contributions presented in the study are included in the article/supplementary material, further inquiries can be directed to the corresponding author.

## Ethics statement

The requirement of ethical approval was waived by Guangdong Second Provincial General Hospital for the studies on humans because this study was a retrospective analysis of previous clinical data. The studies were conducted in accordance with the local legislation and institutional requirements. Written informed consent for participation was not required from the participants or the participants’ legal guardians/next of kin in accordance with the national legislation and institutional requirements. The human samples used in this study were acquired from previous clinical examination and treatment, which had a written informed consent. Written informed consent was obtained from the individual(s) for the publication of any potentially identifiable images or data included in this article.

## Author contributions

Y-lZ: Data curation, Investigation, Methodology, Writing – original draft. Z-sS: Conceptualization, Data curation, Writing – review & editing. W-jH: Data curation, Investigation, Writing – original draft. YC: Data curation, Writing – original draft, Investigation. Y-fZ: Data curation, Writing – review & editing. S-kL: Conceptualization, Methodology, Supervision, Writing – review & editing.
